# Supplementary material for: A modified weighted log-rank test for confirmatory trials with a high proportion of treatment switching
Source: PLoS One. 2021 Nov 15;16(11):e0259178. doi: 10.1371/journal.pone.0259178 (PMC8592474; doi:10.1371/journal.pone.0259178)

Supplementary material for "A modified weighted log-rank test for confirmatory trials with a high proportion of treatment switching"

José L. Jiménez[1], Julia Niewczas[2], Alexander Bore[2], Carl-Fredrik Burman[2]

[1] Global Drug Development, Novartis Pharma A.G., Basel, Switzerland

[2] Statistical Innovation, Data Science & AI, AstraZeneca R&D Gothenburg, Sweden

Additional simulations

S1 **Fig**: Relative efficiency between the modified weighted log-rank ($mWLR$) test and the log-rank (LR) test assuming a fixed value of $m_0^{\text{OS}} = 10$, a value of $m_0^{\text{PFS}} = 1$ month, and varying both p and p' between 0 and 1. Values above 100% favor $mWLR$ and values below 100% favor LR.

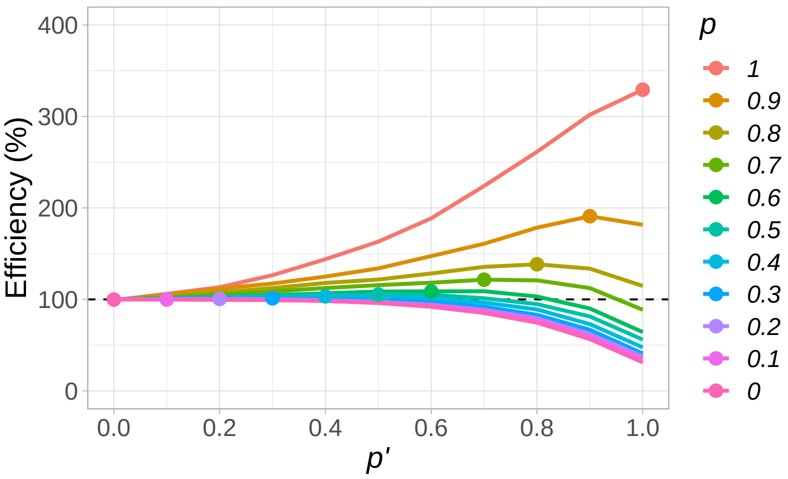

S2 **Fig**: Relative efficiency between the modified weighted log-rank ($mWLR$) test and the log-rank (LR) test assuming a fixed value of $m_0^{\mathrm{OS}} = 10$, a value of $m_0^{\mathrm{PFS}} = 4$ months, and varying both p and p' between 0 and 1. Values above 100% favor $mWLR$ and values below 100% favor LR.

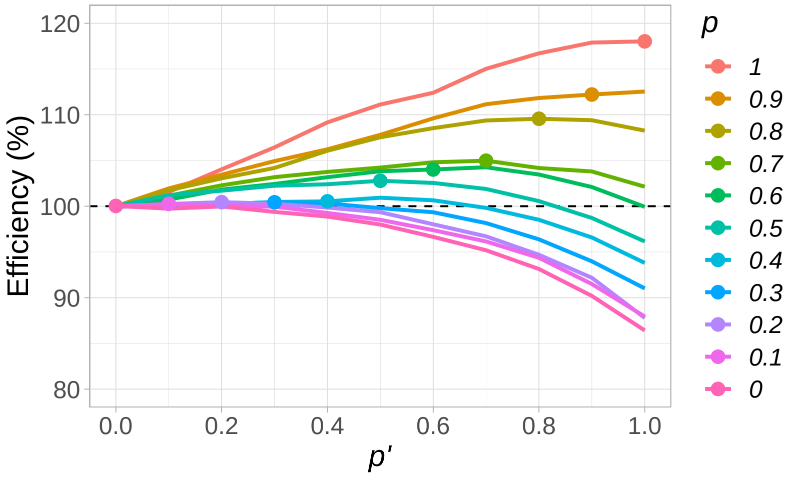

One question one may ask is how our proposed method performs when the underlying time-to-event distribution (i.e., the distribution we use to generate the time-to-event data) is not exponential. In Figure S3 we present an assessment of the power when the time-to-event data is generated with a Weibull distribution with probability density function

$$f(t, \lambda, k) = \begin{cases} \frac{k}{\lambda}\left(\frac{t}{\lambda}\right)^{k-1} \exp(t/\lambda)^k & \text{if} \quad t \geq 0 \\ 0 & \text{if} \quad t < 0 \end{cases} \tag{1}$$

and with median equal to $\lambda(\ln 2)^{1/k}$. We assume that $p = p' = 1$. The value of λ in the experimental group is chosen so that the median OS is equal to 15 months. In the control group, we range the median OS from 5 to 10 months. We assume a median PFS of 2 months for both the experimental and control groups. Of course, to calculate λ we need to provide a value of k. In this assessment, we test values of k that range from 0.5 to 1.5, including $k = 1$ which reduces the Weibull distribution to an exponential distribution. We observe that $mWLR$ performs slightly worse than LR for values of $k \leq 0.7$. In contrast, when $k > 0.7$, our proposal outperforms LR. In the Weibull distribution, when $k < 1$ the hazard function decreases whereas when $k > 1$ the hazard function increases. This does not happen with the exponential distribution, where the hazard function is constant. These results show that out proposal is sensitive to increments/decrements of the hazard function of each arm. Thus, a careful evaluation of the expected hazard function of each treatment group to decide whether the methodology we employ here should be used or not.

S3 **Fig**: Power of the $mWLR$ and LR tests with time-to-event data generated from a Weibull distribution under different values of k.

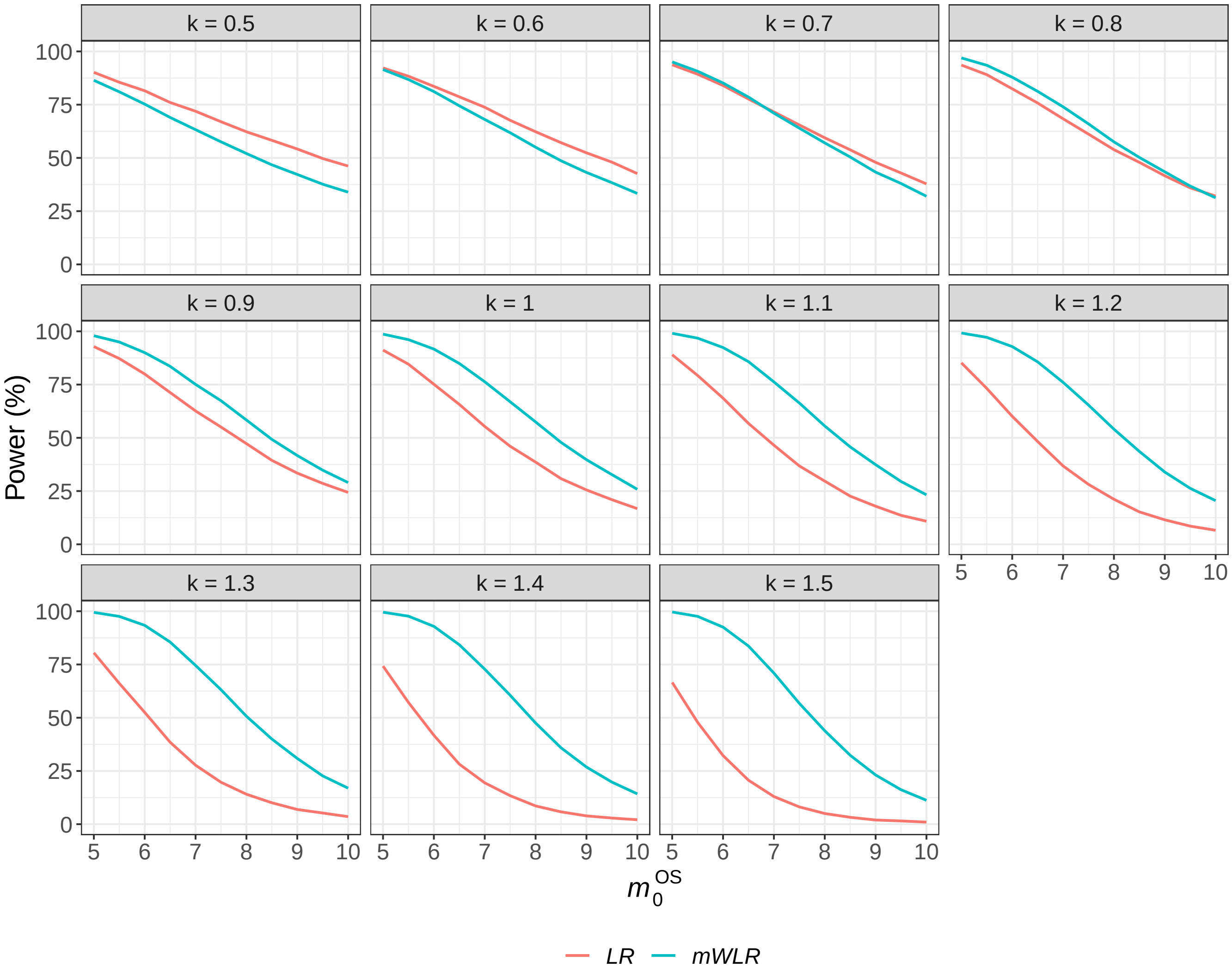

Supplement: S1 File — (PDF) [file pone.0259178.s001.pdf]
