# Supplementary material for: A modified weighted log-rank test for confirmatory trials with a high proportion of treatment switching
Source: PLoS One. 2021 Nov 15;16(11):e0259178. doi: 10.1371/journal.pone.0259178 (PMC8592474; doi:10.1371/journal.pone.0259178)
Supplement: Attachment — Submitted filename: response_to_reviewers.pdf [file pone.0259178.s003.pdf]

Response to reviewers' comments on "A modified weighted log-rank test for confirmatory trials with a high proportion of treatment switching"

José L. Jiménez, Julia Niewczas, Alexander Bore & Carl-Fredrik Burman

September 2021

Dear Reviewers and Associate Editor,

Please find below our responses to your comments. Thank you for your detailed review which has improved the quality and clarity of the paper. All changes are highlighted in blue in the manuscript.

José, Julia, Alexander and Caffe.

Reviewer 1

Q1. How about an adaptive design? (to i.e. assume another underlying distribution or amount of treatment switching)

We thank the reviewer for this comment. This is a relevant point as the amount of treatment switching and the underlying distributions are not fully known when designing the trial. Therefore, it could therefore be of interest to adapt the trial so that such features are reflected in the analysis. One possibility is to consider several test statistics, corresponding to different values of the treatment switching parameter in our model. The p-value will then need to be adjusted based on the dependence between the test statistics. Our preference in a confirmatory trial, however, is to pre-specify the analysis including this parameter best on best guess estimates. The test will still control the type-I error. We believe this could be a nice extension to this article, and thus we add a sentence to the discussion section highlighting it.

Q2. What about the robustness against the assumed underlying distribution (i.e. exponential distribution might often not be given) did you consider Weibull, Gompertz, log-normal distributed data?

We thank the reviewer for this commentary. Indeed, the exponential distribution assumes a constant hazard function over time for each of the treatment groups. We have assessed the performance of our proposal in a setting where time-to-event data is generated from a Weibull(k, λ) distribution. We know from the literature that $k < 1$ causes a decrease in the hazard function and $k > 1$ causes an increase in the hazard function. In fact, results show that standard log-rank test outperforms our proposal if $k \leq 0.7$ (i.e., when there is a decrease in the hazard function of the treatment groups) whereas our proposal outperforms standard log-rank test of $k > 0.7$ (i.e., when there is an increase in the hazard function of the treatment groups). Therefore, we conclude that out test is sensitive to the underlying time-to-event distribution. We believe that with both the exponential and the Weibull distributions we cover the constant hazard function, increasing hazard function and decreasing hazard function settings, and that we have identified strengths and weaknesses of our method. Moreover, we provide the R code to run a similar simulation in case the clinical team believes that their data follows a distribution different from the exponential or Weibull distribution. We have added these results to the supplementary material and we have also added a detailed description of these finding to the main manuscript both in the "robustness" and "discussion" sections.

Q3. What about a situation of crossing hazards?

We thank the reviewer for this comment. In our setup, the hazard functions for the two arms will not be proportional due to treatment switching. There are other factors, such as time-varying treatment efficacy or population heterogeneity, that could lead to non-proportional hazards. Survival curves may even be crossing if one treatment (e.g.

chemotherapy) is effective early, while another (e.g. immune-oncology treatment) has better efficacy long-term. Such sources of non-proportional hazards are outside the scope of this paper. However, we believe that other readers may have the same question and hence we have added to the introduction section a list of useful references that interested readers could use as a starting point.

Q4. Which effect measure is best to go along with your test? What (time-dependent?) effect measure would you propose?

We thank the reviewer for this comment since, apart from showing that two treatment groups are statistically different, we need to estimate the treatment to assess the clinical benefit patients are obtaining. It is well known that the standard logrank test is the most common survival analysis test for confirmatory clinical trials. The test is directly linked to an estimate and a confidence interval for the hazard ratio (HR). It is useful, and common practice in clinical publications, to also report other supporting estimates as the median survival and the entire Kaplan-Meier curves. Especially when hazards are non-proportional, there are issues with using HR as an estimand and period-specific HRs may be mis-leading (Hernán (Epidemiology, 2010); Bartlett et al (2020)). This debate is complicated and we are choosing not to dive into the details as we believe it is out of the scope of this manuscript. Strictly speaking, our method corresponds to a HR estimate, with the correspondent confidence interval. However, many other authors have questioned the clinical interpretation of HR because treatment switching will bias the estimate in a conservative way (i.e., as patient start to switch from placebo to treatment, any treatment effect will progressively disappear). Therefore, we may consider other estimates such as the robust milestone (or landmark) analysis or the RMST difference as simple and clinically interpretable alternatives to the HR. We have modified the paragraph in the discussion where we talk about treatment effect estimates (lines 530-542) to point this out.

Q5. Did you consider what estimand framework the in the your paper described setting/test would refer to?

We thank the reviewer for this comment. We have carefully though about this point and we prefer not to mention the estimand framework in the paper as we don't think this will enhance the understanding of the general problem and methodology we propose in this article. In a practical situation, the target study population has to be carefully defined. This is independent from the problem discussed in the manuscript; we will have the same type of problem for very different populations. There is debate about what is a good estimand for time-to-event trials. This has partly been addressed (see response to the previous comment number 4). The main estimate is typically the HR. However, this may not be what is of greatest relevance to the patient or prescribing physician. The patient will likely not ask "Given that I live today and I had lived today if I had received standard-of-care instead, how much is the risk of dying tomorrow reduced by receiving the new drug?" More reasonable questions would be "What is the chance that I live to see my first grand-child, expected in December?" or in more mathematical terms "What is my 6-month (or 1y, 2y etc) survival? Perhaps "What is my median (mean?) survival? Still, (weighted) logrank tests are generally clearly more powerful than tests directly linked to estimands of greater importance to the patient (mOS, RMST, landmark). One may therefore distinguish between a "testimand", the entity linked to an efficient test, and clinically relevant estimands. One may however disregard this reasoning and state that HR the primary. The intercurrent events that we focus on this manuscript are treatment switching. Given that we focus on HR, we would be interested if the HR if no treatment switching would occur. This HR can be estimated in our kind of set-up. However, while our test is robust against model mis-specifications – it will strongly control the Type 1 Error even if the treatment switching pattern or conditional survival distributions are very different – an attempt to give an unbiased estimate of HR while not disregarding a lot of information after treatment switching will be heavily dependent on assumptions. Thus, this is not suitable as the primary analysis for a trial with regulatory approval intention.

Q6. In formula (1) (test hypothesis): Where is $S_0(t) > S_1(t)$ included? (Not clear whether you have a one or two-sided hypothesis)

Thank you for this question. We have clarified that we have a one-sided test.

Reviewer 2

It is well-written paper and topic is also very important. The authors proposed an alternative weight method for WLR and showed the advantage over existing methods by simulation study. A motivative example is re-analyzed

and demonstrate the potential usefulness of the new proposed method. The idea is clearly explained.

Q1. The simulations are done only with the case matching to the motivation example. I am not sure if the method is still working better for other situations. Maybe additional simulation can help to identify when the mWLR has more advantage.

We thank the reviewer for this comment. We have developed this method with the particular setting we describe in the manuscript (i.e., non-proportional hazards induced by treatment switching) in mind, and we believe that it cannot be applied directly as it is to other non-proportional hazards sources. In the simulations we have done, we cover the correct specification of the hazard rate function, the misspecification of the hazard rate function, and the misspecification of the underlying time-to-event data distribution (as a suggestion from Reviewer 1). With all these results, we are confident that we have identified (and reported) both the strengths and weaknesses of our proposal, with clear statements that indicate when our method could be used and when it shouldn't be used.

Q2. The efficiency measure in formula (21) is the squared ratio between two statistics. Is there a reason/literature to choose this efficiency (why squared)? Is the ration of two powers more natural?

We thank the reviewer for this comment. We have updated equation (21) in the manuscript to be

$$Efficiency_{LR}^{mWLR} = \left(\frac{\Phi^{-1}(1-\alpha) + \Phi^{-1}\left(Power^{mWLR}\right)}{\Phi^{-1}(1-\alpha) + \Phi^{-1}\left(Power^{LR}\right)} \right)^2,$$

which is the one mentioned by the reviewer. In the first version of this manuscript, we based the definition of the relative efficiency on the publication from Burman, Hamren & Olsson (Pharm Stat 2005; section 2.3). It is possible to show that the original and updated definitions are equivalent. However, we are on board with the idea that including the power in the equation may be more natural and hence readers may be more familiar with it.

Minor points:

Q3. The asymptotic property of WLR is fine with fixed weights while mWLR used the weights related to the outcome, the asymptotic property still holds true?

We thank the reviewer for this question. The asymptotic property of WLR would also hold with our proposed mWLR because the weights are fixed prior to the beginning of the study and represent the expected hazard rate function. The idea of this manuscript is to illustrate how a trial could be designed using the mWLR when treatment switching is expected. To do so, we design a hypothetical new clinical trial similar to the RECORD-1 trial, and thus the actual RECORD-1 trial would be the previous study we use to build the expected hazard ratio function. Nevertheless, the key message is that we assume to have this prior knowledge coming from other previous studies, not from the study we design itself. We believe this is well stated in lines 252-263 and 276-279 of the updated manuscript.

Q4. Figure 1, it is better to put sample sizes for plot A, B, C in figure's caption.

We thank the reviewer for this comment. We have added the sample sizes to the caption of the Figure.
